# Impact of atrial tachyarrhythmias on paravalvular regurgitation post-transcatheter aortic valve implantation: recognition and management

**DOI:** 10.1093/ehjcr/ytae346

**Published:** 2024-07-19

**Authors:** Jingyao Yang, Yu Du, Yujie Zhou, Zhijian Wang

**Affiliations:** Department of Cardiology, Beijing Anzhen Hospital, Capital Medical University, Beijing 100029, China; Beijing Institute of Heart Lung and Blood Vessel Disease, Beijing Key Laboratory of Precision Medicine of Coronary Atherosclerotic Disease, Clinical Center for Coronary Heart Disease, Capital Medical University, Beijing 100029, China; Department of Cardiology, Beijing Anzhen Hospital, Capital Medical University, Beijing 100029, China; Beijing Institute of Heart Lung and Blood Vessel Disease, Beijing Key Laboratory of Precision Medicine of Coronary Atherosclerotic Disease, Clinical Center for Coronary Heart Disease, Capital Medical University, Beijing 100029, China; Department of Cardiology, Beijing Anzhen Hospital, Capital Medical University, Beijing 100029, China; Beijing Institute of Heart Lung and Blood Vessel Disease, Beijing Key Laboratory of Precision Medicine of Coronary Atherosclerotic Disease, Clinical Center for Coronary Heart Disease, Capital Medical University, Beijing 100029, China; Department of Cardiology, Beijing Anzhen Hospital, Capital Medical University, Beijing 100029, China; Beijing Institute of Heart Lung and Blood Vessel Disease, Beijing Key Laboratory of Precision Medicine of Coronary Atherosclerotic Disease, Clinical Center for Coronary Heart Disease, Capital Medical University, Beijing 100029, China

A 70-year-old woman with severe aortic stenosis was admitted for transcatheter aortic valve implantation (TAVI). The patient had a type I bicuspid aortic valve with right–left cusp fusion. Intra-operative transthoracic echocardiography (TTE) and aortography after prosthesis (Taurus NXT#26, Peijia Medical) implantation showed mild aortic paravalvular regurgitation (PVR) (*Figure 1A–C*; Supplementary material online, *Videos S1–S3*), while the electrocardiogram showed atrial tachyarrhythmias. However, after removing the left ventricular guidewire and femoral artery sheath, repeat TTE showed significant deterioration in the prior PVR (*Figure 1D* and *E*; Supplementary material online, *Videos S4* and *S5*), when the patient was in sinus rhythm with a normal heart rate. Therefore, re-access via femoral artery was performed, followed by aortography confirming a moderate-to-severe PVR from aortic right–left cusp fusion (*Figure 1F*; Supplementary material online, *Video S6*). After implanting a second prosthesis (Taurus NXT#26, Peijia Medical), TTE showed trace PVR while atrial tachyarrhythmias recurred (*Figure 1G*; Supplementary material online, *Video S7*). To ensure accurate assessment of PVR, propafenone 70 mg was administered intravenously. Finally, both the intra-operative TTE and aortography showed trace PVR (*Figure 1H* and *I*; Supplementary material online, *Videos S8* and *S9*) when the patient was in normal rhythm and heart rate.

**Figure 1:**
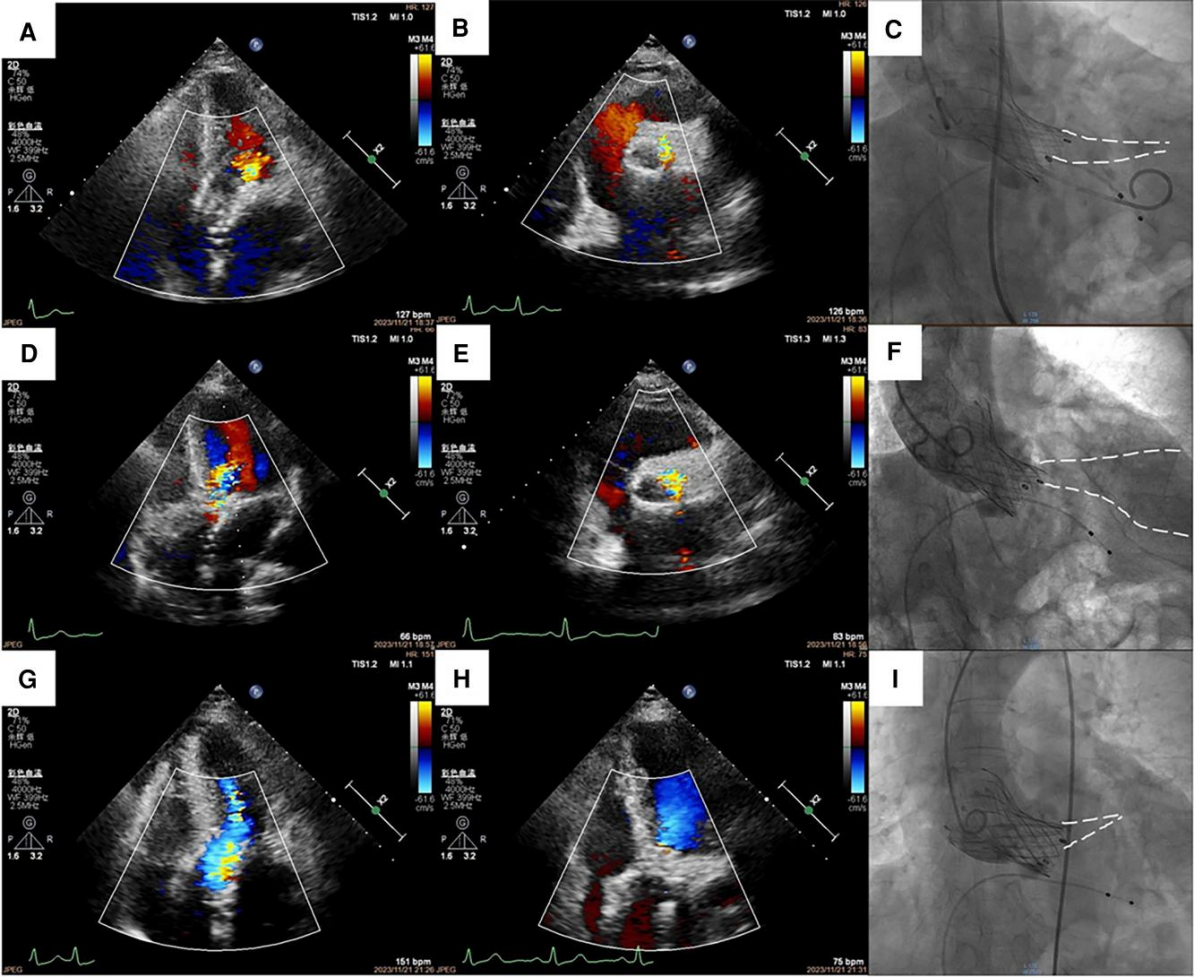
Intra-operative evaluation of aortic paravalvular regurgitation using transthoracic echocardiography and aortography. Patient with atrial tachyarrhythmias, apical five-chamber view (*A*; Supplementary material online, *Video S1*) and parasternal short-axis view (*B*; Supplementary material online, *Video S2*) on transthoracic echocardiography and cusp-overlap view on aortography (*C*; Supplementary material online, *Video S3*) showing mild paravalvular regurgitation after first prosthesis implantation. After the patient restoring normal haemodynamics, apical five-chamber view (*D*; Supplementary material online, *Video S4*) and parasternal short-axis view (*E*; Supplementary material online, *Video S5*) on transthoracic echocardiography and cusp-overlap view on aortography (*F*; Supplementary material online, *Video S6*) showing moderate-to-severe paravalvular regurgitation. After valve-in-valve procedure, apical five-chamber view (*G*; Supplementary material online, *Video S7*) showing trace paravalvular regurgitation while atrial tachyarrhythmia reoccurring. After achieving normal rhythm and heart rate using propafenone, apical five-chamber view (*H*; Supplementary material online, *Video S8*) on transthoracic echocardiography and cusp-overlap view (*I*; Supplementary material online, *Video S9*) on aortography confirming trace paravalvular regurgitation. White dash line in *C*, *F*, and *I* indicated the border of paravalvular regurgitation on aortography.

Evaluation of PVR during TAVI procedure using TTE relies heavily on colour Doppler jet characteristics, easily affected by abnormal haemodynamics (i.e. arrhythmias).^1^ To ensure an accurate assessment of PVR, pharmacological stabilization of haemodynamics is indispensable, and integration of other echocardiographic and invasive haemodynamic findings is also helpful.

## Supplementary Material

ytae346_Supplementary_Data

## Supplementary material

**Consent:** The authors certify that they have obtained the patient’s consent to the publication of information about her in a journal or thesis.

**Funding:** This work was supported by Beijing Municipal Natural Science Foundation, General Program (7232039), National Natural Science Foundation of China, General Program (82370449), and The Capital Health Research and Development of Special (2022-2-1052).

**Data availability:** The data underlying this article will be shared on reasonable request to the corresponding author.
